# Maternal obesity programs cardiac remodeling in offspring via epigenetic, metabolic, and immune dysregulations

**DOI:** 10.1101/2025.04.15.648971

**Published:** 2025-05-27

**Authors:** Elysse A. Philips, Yem Alharithi, Tim D. Wilson, Cameron Broberg, Brett A. Davis, Sheryl Koch, Lucia Carbone, Jack Rubinstein, Susan B. Gurley, Sandra Rugonyi, Sushil Kumar, Alina Maloyan

**Affiliations:** 1 Department of Medicine, Knight Cardiovascular Institute, Oregon Health & Science University, Portland, OR, 97239; 2 Department of Molecular and Medical Genetics, Oregon Health & Science University, Portland, OR, 97239; 3 Division of Cardiovascular Health and Disease, Department of Internal Medicine, College of Medicine, University of Cincinnati, Cincinnati, OH, 45221; 4 Division of Genetics, Oregon National Primate Research Center, Beaverton, OR, 97315; 5 Department of Medicine, University of Southern California, Los Angeles, CA; 6 Department of Biomedical Engineering, Oregon Health & Science University, Portland, OR, 97239; 7 Department of Cell, Development and Cancer Biology, Knight Cancer Institute, Oregon Health & Science University, Portland, OR, 97239

**Keywords:** maternal obesity, developmental programming, cardiac epigenetics, inflammation

## Abstract

Maternal obesity during pregnancy significantly increases the offspring’s risk of later-life cardiovascular disease. This study investigated cardiometabolic perturbations by utilizing a mouse model of maternal high-fat diet (HFD)-induced obesity that recapitulates metabolic abnormalities observed in humans. We report that offspring of HFD-fed mothers (Off-HFD) exhibit a progression of obesity, hypertension, dyslipidemia, and metabolic inflexibility when compared with offspring of regular diet-fed mothers (Off-RD). Deeper investigation of cardiac function further identified significant functional, metabolic, and immune perturbations in adult offspring of mothers on HFD. Specifically, Off-HFD mice presented progressing cardiac hypertrophy with reduced ejection fraction, increased accumulation of fibrotic tissue, mitochondrial dysfunction, and altered immune complexity including increases in cardiac resident and infiltrated macrophages, and decreases in CD4+ and CD8+ T-cell subpopulations. While these alterations may not be immediately catastrophic, they likely predispose the offspring to heightened sensitivity to nutritional, psychological, or environmental stressors. Analysis of DNA methylation in the hearts of newly-weaned offspring from RD and HFD mothers revealed numerous differentially methylated CpGs and regions within genes associated with cardiac development, hypertrophy, mitochondrial function, and immune response. Thus, our study shows epigenetic remodeling early in development, which is likely responsible for the cardiovascular dysregulation observed in adult life. These findings uncover potential windows of opportunity for preventive therapy and early therapeutic interventions.

## INTRODUCTION

Obesity is a rapidly growing health concern; in the last 30 years, its presence in the United States has doubled(1). In 2022, the worldwide prevalence of obesity among adults was 16% (890 million), with 43% (2.5 billion) being overweight (2). Similarly, the prevalence of overweight and obesity among women of reproductive age (15–49 years old) has increased from 28.4% in 1999–2000 to 41.5% in 2016 (3) and is becoming a serious public health issue. Importantly, obesity promotes cardiovascular disease via dysregulations in endothelial function and vascular remodeling that cause atherosclerotic and vasospastic coronary heart disease, arrhythmias, cardiomyopathy, and congestive heart failure (2, 4). In the pregnancy context, both pregestational obesity and greater than healthy gestational weight gain complicate pregnancy *per se* and significantly increase the risk of adverse outcomes (2). Moreover, obesity in pregnancy programs the offspring to become obese and to develop later-life cardiovascular and metabolic diseases, including Type II diabetes (5–9). Thus, maternal obesity is a key element in a positive-feedback loop, initiating a vicious intergenerational cycle of obesity that cannot be explained by genetics or lifestyle choices (10, 11).

A typical pregnancy depends on the tightly-regulated transfer of nutrition to the fetus, which is governed through extensive fetal-maternal communication. Consequently, alterations in maternal health and perturbation of that communication leave long-lasting imprints on the fetus, an effect known as developmental programming, that can result in structural and physiological abnormalities that present as disease later in the offspring’s life. Epidemiological data link obesity in pregnancy with major birth defects, including neural tube and cardiac defects (12), and with increased risk of neurodevelopmental disorders, including cerebral palsy, attention-deficit disorder, cognitive delay, and even autism (13–15). Likewise, there is a clear epidemiologic correlation between increased maternal BMI and offspring cardiovascular disease (2, 16). A systematic review of five studies assessing the relationship between maternal obesity and congenital heart diseases found a significant correlation (17), and another review of 14 studies found a dose-response effect between maternal weight and offspring heart disease (18). More broadly, children born to obese mothers present structural and functional cardiovascular dysregulations that increase the likelihood of later-life cardiac events and premature mortality (5, 19–28). Thus, early-life adversity stemming from maternal obesity establishes a susceptibility to cardiovascular disease throughout life.

Building on our established mouse model of maternal high-fat diet (HFD)-induced adiposity, which replicates metabolic abnormalities seen in offspring of obese mothers (including obesity, glucose intolerance, and asthma (29–31)), this study tested the hypothesis that maternal HFD independently drives offspring cardiovascular dysfunction, separate from the influence of adverse postnatal lifestyle factors. We further examined the effects of maternal HFD on offspring cardiac function, epigenetics, metabolism, and immune complexity.

## MATERIALS AND METHODS

### Study approval.

All animal experiments were approved by Oregon Health & Science University’s Institutional Animal Use Committee (IACUC protocol # IP00432).

### Antibodies.

Total OXPHOS Rodent Western Blot Antibody Cocktail was purchased from Abcam (Waltham, MA, cat # ab110413). Antibodies against Vinculin, p62 and Rubicon were purchased from Cell Signaling (Danvers, MA, cat # 4650, 5114 and 8465 respectively). Anti-DGAT1 antibody was purchased from Proteintech (Rosemont, IL, cat # 11561–1-AP), and the antibody against autophagy regulator TFEB was purchased from Invitrogen (Waltham, MA, cat #PA1–31552).

### Mouse model of maternal obesity.

All mouse studies were done using wild-type FVB/N mice. Mice were kept under a 12-hour light/dark cycle in temperatures between 18–23 degrees Celsius with 40–60% humidity, in stress-free/bacteria-free conditions. Mice were caged in groups of three to five whenever possible. Food and water were given ad libitum, and body weight was determined weekly. To induce maternal obesity, a high-fat diet (Teklad Cat#TD.06415) or its control, a regular diet (RD, PicoLab^®^ Laboratory Rodent Diet Cat # 5L0D), was given to virgin female FVB/NJ mice from six weeks of age and throughout the entire study. The diet compositions have been reported before (32) After eight weeks of dietary intervention, RD- and HFD-fed female mice were bred to an age-matched RD-fed male. Offspring were fed the RD only starting from weaning and throughout their life.

### Metabolic phenotyping.

Oxygen consumption and respiratory exchange ratio (RER) were measured in individually housed mice by Vanderbilt University’s Mouse Metabolic Phenotyping Core using the Promethion from Sable Systems (Las Vegas, NV).

**Mean arterial pressure** was non-invasively measured using a CODA^®^ tail-cuff blood pressure system (Kent Scientific, Torrington, CT) after two weeks of training.

**Blood lipid profiling** was performed at the Mouse Metabolic Phenotyping Center of the University of Cincinnati. Plasma triglycerides were measured using the Randox Triglyceride Assay Kit (Randox Laboratories, Kearneysville, WV). Plasma cholesterol levels were determined using the Infinity Total Cholesterol Assay Kit (Fisher Scientific, Waltham, MA). Plasma phospholipids were assessed using the Phospholipids C Assay (Wako Life Sciences, Inc., Mountain View, CA). Plasma non-esterified fatty acids were measured using the Wako HR Series NEFA-HR (2) (Wako Life Sciences, Inc., Mountain View, CA). All measurements were conducted according to the manufacturer’s instructions.

### Transmission electron microscopy.

Hearts were perfused with 3.5% glutaraldehyde in 0.1 M/L phosphate buffer, pH 7.4 and embedded in Embed 812 resin (Electron Microscopy Science) for sectioning. The ultrathin sections were visualized by the Multiscale Microscopy Core at OHSU using a FEI Tecnai^™^ with iCORR^™^ transmission electron microscope (Thermo Fisher Scientific, Hillsboro, Oregon).

### Protein isolation.

Proteins were isolated from mouse hearts and lysed using ice-cold radioimmunoprecipitation assay buffer with freshly-added protease and phosphatase inhibitors. The resulting mixture was transferred to a 1.5 ml tube and centrifuged at 1000 x *g* for ten minutes at 4 °C to remove cellular debris. The supernatant was then transferred to a fresh tube and protein concentration quantified using a Pierce Bicinchoninic Acid Protein Assay Kit (Thermo Fisher Scientific, Waltham, MA).

### Western blotting.

Myocardial proteins (10–25 mg) were separated on a 4–20% SDS-PAGE gel, then transferred to a polyvinylidene difluoride membrane and blocked for one hour in 5% (w/v) milk in TBS solution with 0.1% Tween 20. Membranes were subsequently incubated overnight with primary antibodies, washed, blocked in 1% milk, and probed with secondary antibodies conjugated to HRP. Western blot membranes were visualized using G:Box and analyzed using GeneTools, both utilities from Syngene (Frederick, MD). All samples were normalized to housekeeping genes.

### RRBS method and analysis.

DNA methylation was evaluated using reduced representation bisulfite sequencing (RRBS) (33), a genome-wide approach that allows the capture of key regulatory regions including promoters, CpG islands, and CpG island shores. RRBS libraries were generated by the Knight Cardiovascular Institute Epigenetics core using established methods (34, 35). Briefly, genomic DNA from left ventricle samples were digested overnight with *Msp*I (New England Biolabs) to produce sticky ends starting with a CpG. Libraries were then prepared using the NEXTflex Bisulfite-Seq Kit (BioScientific) and bisulfite conversion was performed with the EZ DNA Methylation-Gold Kit (Zymo Research). After quality control, libraries were multiplexed and sequenced on a NovaSeq 6000 at the OHSU Massively Parallel Sequencing Shared Resource to obtain roughly 40 million reads/library. Sequencing data were downloaded and analyzed as described in Carbone et al. (34). Briefly, after evaluation with FastQC (Andrews, 2010. http://www.bioinformatics.babraham.ac.uk/projects/fastqc), read alignment to the mouse reference genome (mm10) and methylation calling on every covered cytosine were performed with Bismark (36). Coverage files from Bismark methylation extractor were used to obtain the coverage and methylation rate of each covered CpG. A methylation rate table was constructed to include only CpGs with minimum 10X coverage and found on canonical chromosomes (1–19 and X,Y). For differential analysis, the table was subset to include CpGs with at least 10X coverage in at least 2 replicates per group of maternal diet (High Fat Diet or Regular Diet) resulting in 421,177 CpGs. Limma (37) was used for differentially methylated cytosine (DMC) analysis. To stabilize variance and improve normality, methylation percentages were transformed using the **arcsin square root transformation**. A linear model was fitted with sample groups and sex as covariates, and empirical Bayes moderation was applied to estimate DMCs. Statistical significance was determined using **moderated t-tests**, and multiple testing correction was performed using the **Benjamini-Hochberg false discovery rate (FDR)** method. Comb-p (38) was used for differentially methylated region (DMR) analysis, using the p-values obtained from DMC analysis as input. The following parameters were used with comb-p pipeline: -c 4 --dist 300 --seed 0.05 --step 60. Genes overlapping or close to DMRs were annotated using custom scripts, and pathway enrichment analyses were conducted using publicly available tools (e.g. GREAT, *EnrichR*) (39, 40). To examine potential transcription factors involved in the dysregulation of key genes/pathways, we used Hypergeometric Optimization of Motif EnRichment (HOMER) (41) to detect enriched binding motifs in the putatively functional DMRs.

To visualize sample similarity in terms of methylation profiles, we performed principal component analysis on the methylation percentages of 343,296 CpGs with at least 10X coverage; the resulting plot is presented in Supplemental Figure 1. One male Off-HFD sample was excluded due to low coverage. A few samples appeared to segregate away from the cluster formed by the rest; these few also showed the highest methylation in non-CpG contexts. When those segregated samples were excluded, the plot showed female Off-RD to cluster with female Off-HFD, and males to similarly cluster together (Supplemental Figure 1).

### Gene Ontology and pathway analysis.

Enrichment analysis of Gene Ontology (release date 20210101) terms was performed for genes associated with differentially methylated CpGs (DMCs) and DMRs using Panther (release date 20200728). Hypo- and hypermethylated regions were jointly analyzed for overrepresentation using Fischer’s exact test with FDR correction (FDR<0.05), with fold enrichment of over- and underrepresented pathways reported relative to the whole mouse genome (which was used as the reference list) (33). Pathway analysis of DMR genes was conducted using Ingenuity Pathway Analysis (Qiagen, Redwood City, CA) and STRING (Version 12.0).

### Transcription factor binding site analysis.

Enrichment of transcription factor binding sites within DMRs was performed using HOMER (41).

### Echocardiography.

Mice were anaesthetized with isoflurane and subjected to echocardiography at the Small Animal Research Imaging Center at OHSU using Vevo LAB 3.1.1 (FUJIFILM VisualSonics, Inc.). Cardiac function and structure values were obtained from B mode images from the parasternal long axis (PSLAX). Left ventricular diameter (LVID), posterior wall (LVPW) and intraventricular septum (IVS) were measured in both systole (s) and diastole (d). B-mode PSLAX were used to calculate ejection fraction (EF), LV mass and stroke volume (SV). Stroke volume was calculated as the product of the left ventricular (LV) outflow tract cross-sectional area and velocity-time integral on angle-corrected pulsed-wave Doppler. LV end-diastolic and end-systolic volumes were calculated from short-axis linear dimensions and long-axis dimensions using established geometric assumptions.

### Western blotting.

Myocardial proteins (10–25 mg) were separated on a 4–20% SDS-PAGE gel, then transferred to a polyvinylidene difluoride membrane and blocked for one hour in 5% (w/v) milk in TBS solution with 0.1% Tween 20. Membranes were subsequently incubated overnight with primary antibodies, washed, blocked in 1% milk, and probed with secondary antibodies conjugated to HRP. Western blot membranes were visualized using G:Box and analyzed using GeneTools, both utilities from Syngene (Frederick, MD). All samples were normalized to housekeeping genes.

### Histology.

H&E and Masson trichrome staining were performed at the OHSU Histopathology Core. Trichrome-stained sections were used to evaluate myocyte cross-sectional area with the aid of ImageJ (National Institutes of Health, Bethesda, Maryland).

### Flow cytometry.

The list of antibodies is presented in Supplemental Table 1. In preparation for flow cytometry, nearly 10^6^ cells from individual hearts were first incubated on ice for 30 min in a solution consisting of 1:10 Fc Receptor Binding Inhibitor (eBiosciences) and 1:500 Live/Dead Aqua stain (Invitrogen) in PBS. Cells were then combined with fluorescently-labeled monoclonal antibodies as previously described (42) in a solution of PBS + 5% FCS + 1.0 mM EDTA (FACS buffer). After another 30 min incubation on ice, the cells were washed 1x with FACS buffer. Next, the stained cells were treated with permeabilization/fixation buffer (eBioscience) on ice for ten minutes, then washed 1x using permeabilization buffer (eBioscience). The permeabilized cells were then subjected to intracellular staining by incubation with fluorescently-labeled monoclonal antibodies in FACS buffer for 30 minutes on ice. Finally, flow cytometry data was acquired on a Cytek Aurora (Cytek Biosciences) spectral flow cytometer and analyzed using FlowJo software v9.5 (Ashland, OR).

### Statistical analysis.

Means were compared using two-way analysis of variance (two-way ANOVA) followed by Student’s *t*-test (corrected for multiple comparisons) or its non-parametrical equivalent. The obtained *p*-values are indicated in each respective graph. Significance was set at an alpha of 0.05. When no sex-dependent differences were found, the results from males and females were pooled.

## RESULTS

### Physiological changes in the mouse offspring of obese mothers.

**Figure 1.**
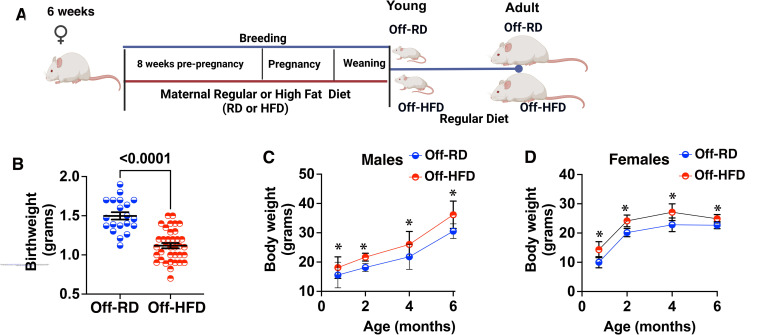
Birthweight- and age-dependent body weight changes in Off-RD and Off-HFD. A, Mouse model of maternal diet-induced obesity. B, Birthweights of Off-RD and Off-HFD mice. C,D, Change in body weights from weaning to six months of age in male (C) and female (D) Off-RD and Off-HFD mice. Data were analyzed by two-way ANOVA followed by multiple unpaired *t*-tests. N= 20–44/group of maternal diet/age. Data are represented as either individual values (B) or as mean±SEM (C-D). *P* values are either shown (B) or labeled with * when *p*<0.05 for Off-HFD vs. Off-RD at each age (C-D).

Six-week-old females were placed on either a regular (13% kcal from fat) or a high-fat (45% kcal from fat) diet (RD and HFD, respectively) (Fig.1A). After eight weeks of dietary intervention, the females were bred to RD-fed males. Birthweights were significantly lower in the offspring of HFD-fed mothers (Off-HFD) when compared with offspring of RD-fed mothers (Off-RD) (Fig.1B). However, despite being born smaller, both male (Fig.1C) and female Off-HFD (Fig.1D) caught up by weaning (3-week-old) and then remained heavier than Off-RD mice throughout their life.

### Whole body metabolic alterations in the offspring of HFD-fed mothers.

**Figure 2.**
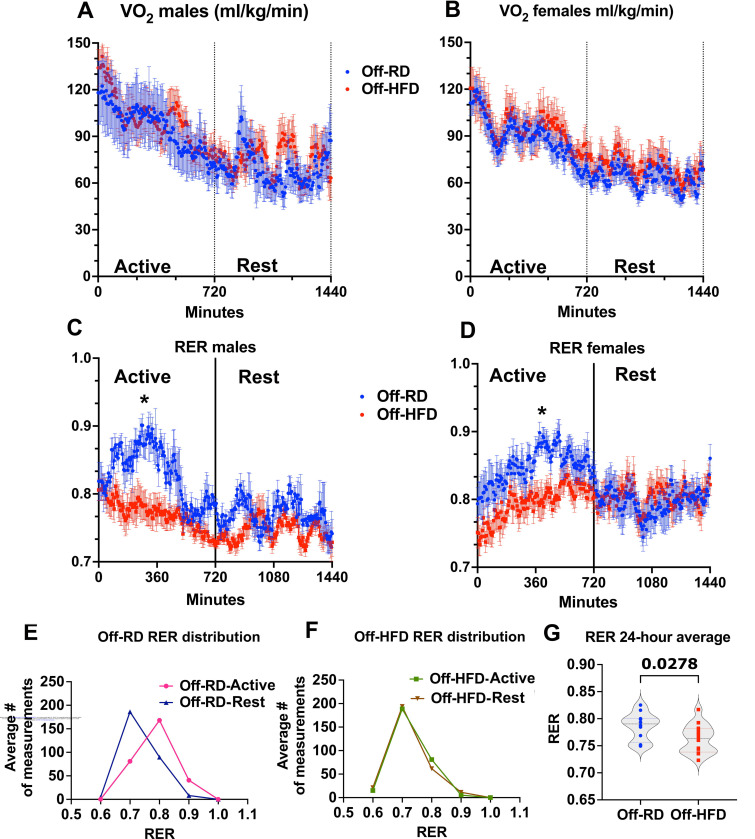
Maternal high-fat diet feeding changes metabolic phenotype in offspring. A-G, Oxygen consumption and respiratory exchange ratio (RER) in four-month-old male and female Off-RD and Off-HFD mice. A-B, Oxygen consumption curves calculated for male (A) and female (B) mice over a 24-hour period (12 hours active and 12 hours rest). C-D, RER in male (C) and female (D) Off-RD and Off-HFD mice over active and rest periods. E-F, Shift in RER between active and rest periods in Off-RD (E) and Off-HFD (F) mice. Male and female data are pooled. *, *p*<0.05 calculated by multiple unpaired *t*-tests with false discovery rate (FDR) <0.01. G, Average 24-hour RER in Off-RD and Off-HFD, male and female data pooled. Data are represented as individual values with lines at mean with SEM; *p*-values are shown, calculated by t-test. N=5–8/group of maternal diet/sex.

Despite the fact that Off-HFD mice show consistent increase in body weight and percentage of fat (29), surprisingly, no between-group differences in food intake and activity levels were observed (32). We next assessed the effects of maternal obesity on whole-body metabolic parameters in adult 16-week-old Off-RD and Off-HFD mice. This assessment revealed no significant changes in the 24-hour pattern of oxygen consumption (Fig.2A–B). However, significant alterations were identified in respiratory exchange ratio (RER, Fig.2C–D). In Off-RD mice, the 24-hour oscillation in RER, an indicator of substrate utilization between active and resting periods, alternated between high values (utilization of carbohydrates) in active periods and low values (utilization of fats) during resting periods (Fig.2C–E). These oscillations were completely abolished in Off-HFD mice (Fig.2C–D, F), suggesting metabolic inflexibility, and daily RER was significantly lower in Off-HFD vs. Off-RD (Fig.2G).

### Cardiovascular and metabolic perturbations in the offspring of HFD-fed mothers.

**Figure 3.**
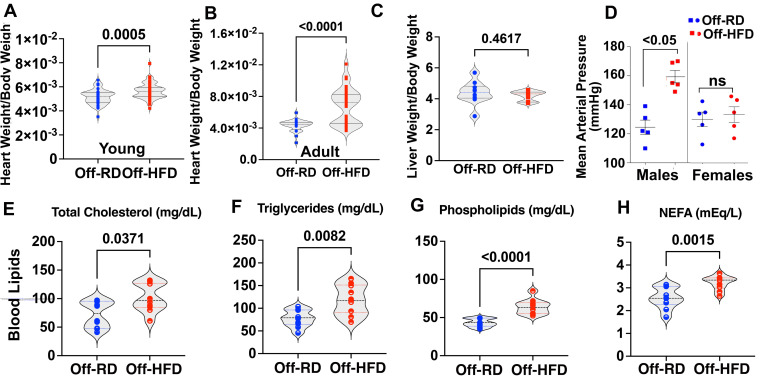
Cardio-metabolic changes in the offspring of HFD-fed mothers. A-B, Heart weight-to-body weight ratio in newly weaned (A) and six-month-old (B) Off-RD and Off-HFD mice fed a normal chow diet. N=38–62 mice/group of maternal diet. C, Liver weight-to-body weight ratio, n=10/experimental group. D, Tail cuff-measured mean arterial pressure in male and female Off-RD and Off-HFD mice, n=5–7 mice/sex/group of maternal diet. E-H, Changes in blood lipids in Off-HFD vs. Off-RD, including total cholesterol (E), triglycerides (F), phospholipids (G), and non-esterified fatty acids (NEFA, H). Data were analyzed by multiple *t*-tests with FDR<0.01. Data are represented as individual values with lines at mean with SEM. N=5/sex/group of maternal diet, 20 animals overall. Data from males and females were pooled. *P*-values are shown.

We next focused on the impact of maternal obesity on cardiovascular remodeling in offspring. At three weeks of age, newly weaned Off-HFD mice showed a moderate (8.6%) increase in heart-weight-to-body-weight ratio as compared with age-matched Off-RD mice (p=0.0005, Fig.3A). By six months of age, Off-HFD mice showed more dramatic 60% increase in heart weight to body weight ratios, indicating a progressive hypertrophy phenotype (Fig.3B). No changes were observed in liver weight-to-body weight ratio in these mice (Fig.3C). We further measured mean arterial pressure in 16-week-old Off-RD and Off-HFD mice using a tail cuff sphygmomanometer, and found a significant increase in male Off-HFD mice but no change in female Off-HFD mice (Fig.3D). Blood lipid profiling revealed significantly increased levels of total cholesterol, triglycerides, phospholipids, and non-esterified fatty acids in both male and female Off-HFD vs. Off-RD, suggesting dyslipidemia (Fig.3E–H).

**Figure 4.**
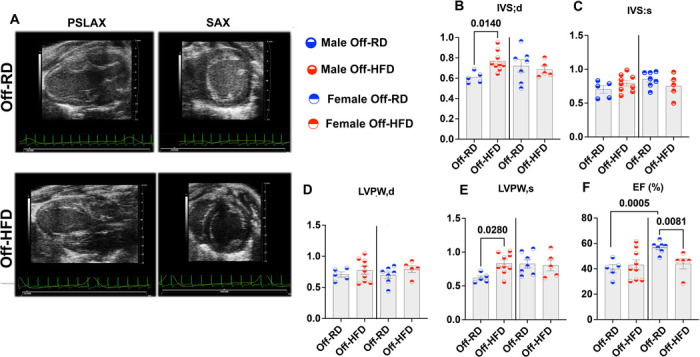
Echocardiographic evaluation of adult male and female Off-RD and Off-HFD mice. A, Representative parasternal long axis B mode images of the left ventricle and short axis. B-F, Sex-dependent changes in diastolic (B) and systolic (C) intra-ventricular septum thickness; and diastolic (D) and systolic (E) left ventricular posterior wall thickness. F, Quantification of left ventricular ejection fraction (EF) expressed in percent (%). N=5–9/sex/group of maternal diet. Data are represented as individual values for males (left) and females (right) with lines at mean with SEM. Significant differences was determined by Mann-Whitney test followed by unpaired *t*-test; p-values are shown.

We then conducted cardiac function assessments in adult 24–30-week-old male and female Off-HFD and Off-RD mice using echocardiography. The majority of echocardiographic parameters were not significantly different between the groups (Supplemental Table 2); however, our data importantly revealed sexual dimorphism in offspring cardiac response to maternal obesity (Figure 4A). Specifically, IVS;d and LVPW;s were increased in male but not female Off-HFD vs. Off-RD (Fig.4B, E), probably due to increased mean arterial pressure (though IVS;s, and LVPW;d trended to be increased as well, neither reached statistical significance(Fig.4C–D)). In contrast, ejection fraction was reduced only in female Off-HFD vs. Off-RD mice (Fig.4F). Notably, the opposite sex-specific difference was observed among Off-RD mice, with male mice having significantly lower ejection fraction than females with these differences being abolished in Off-HFD mice (Fig.4F).

### DNA methylation pattern in left ventricle is affected in newly weaned Off-HFD mice.

Published evidence suggests that prenatal and early postconceptional periods comprise the critical window during which DNA methylation patterns are established (43–46). In light of the observed impacts of maternal obesity on progression of adult cardiovascular disease in offspring, we took a step back to reveal cardiac epigenetic processes that precede cardiovascular dysfunction in Off-HFD mice. To determine regions and individual CpGs with significant differential methylation between offspring of HFD- and RD-fed mothers, we conducted reduced representation bisulfite sequencing (RRBS) on left ventricular samples from newly weaned three-week-old male and female Off-RD and Off-FD. The number of reads obtained per sample before and after trimming is provided in Supplemental Table 3. To visualize sample similarity in terms of methylation profiles, we performed principal component analysis on the methylation percentages of 343,296 CpGs with at least 10 X coverage; the resulting plot is presented in Supplemental Figure 1. One male Off-HFD sample was excluded due to low coverage. Three samples appeared to segregate away from the cluster formed by the rest; these few also showed the highest methylation in non-CpG contexts. When those segregated samples were excluded, the plot showed female Off-RD to cluster with female Off-HFD, and males to similarly cluster together (Supplemental Figure 1).

**Figure 5.**
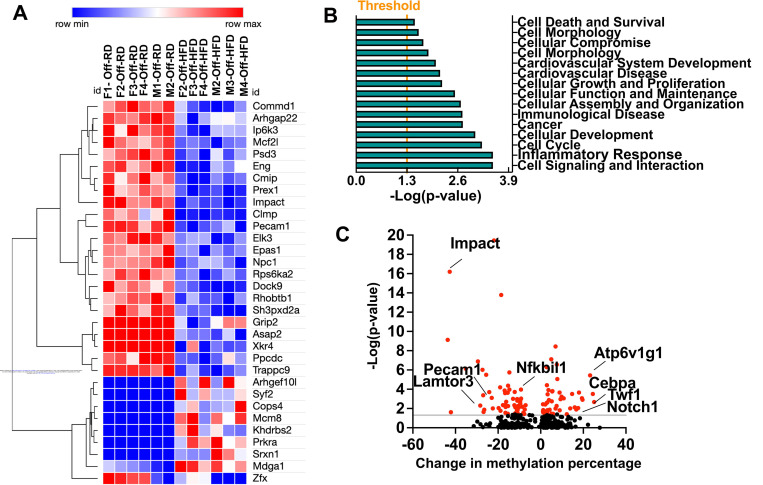
Mapping and comparisons of the DNA methylomes of newly weaned Off-RD and OFF-HFD. A, Heatmap and unsupervised hierarchical clustering of the 12 Off-RD and Off-HFD samples based on individual DNA methylation status. B, Bar graphs showing canonical pathways identified by Ingenuity Pathway Analysis as enriched among differentially methylated genes. C, Volcano plot showing statistical significance (-log(*p*-value)) vs. change in methylation percentage. Several differentially methylated genes with highest amplitudes of change are labeled. Grey line indicates the threshold for significance (-log(0.05)=1.3). Data from males and females were pooled.

Our analysis identified 89 significant DMCs present in both male and female Off-HFD vs. Off-RD (FDR<0.05, *p*-value<0.001, amplitude of change >10%). From those, we identified 32 CpGs that overlapped with annotated genes (Fig.5A). Importantly, our data show that only 22% of the DMCs exhibited increased CpG methylation in Off-HFD mice. The single greatest reduction in CpG methylation (47%) was observed for the gene *Impact*, a transcriptional regulator associated with protein sequestering activity under the stress conditions such as amino acid starvation (47). Meanwhile, two genes tied for the greatest increase in CpG methylation (>20%): Ectonucleotide Pyrophosphatase/Phosphodiesterase 3 (*Enpp3*), an extracellular enzyme that acts as a mediator of cell-cell innate immune communication (48); and Centrosomal Protein 72 (*Cep72*), a component of centriolar satellites previously implicated in mitotic spindle formation (49).

To understand what biological networks in the heart are affected by differential methylation downstream of maternal HFD, we applied the Ingenuity Pathway Analysis software to all genes with DMCs (FDR<0.05, *p*<0.05, absolute differential methylation >10%), genes by direction of change (hyper- and hypo-methylated), and data from both sexes (Fig.5B). We found several networks to be affected by maternal obesity (*p*< 0.05), including Cell Signaling and Interaction, Inflammatory Response, Cell Cycle, Cancer, Cardiovascular System Development, and Immunological Disease.

In the analysis of DMRs, absolute methylation differences >10% were considered significant at a Šídák multiple comparison *p*-value <0.05. Overall, 61 DMRs were identified within protein-encoding genes (Fig.5C), with 13 hypermethylated and 48 hypomethylated. The top ten hyper- and hypomethylated DMRs and associated genes are given in Supplemental Tables 4 and 5. We utilized STRING network analysis to determine which protein interaction networks were most subject to methylation changes in Off-HFD mice. Supplemental Table 6 presents the list of differentially methylated genes specifically involved in cardiovascular development and function. Among the genes with differentially methylated DNA, we observed a significant enrichment of those involved in cardiac morphogenesis and development (*Adamts1, Eng, F2r, Jmjd6, Notch1, Nrap,* and *Nrp2*), cardiac hypertrophy (*Adgrf5, F2r, Twf1*), immune responses including macrophage differentiation and activation (*Adgrf5, Atp6v1g1, Cebpa, Epas1, F2r, Nfkbil1, Notch1, Pecam1,* and *Shb*), mitochondrial function (*Cebpa, Cry1, Epas1*), and autophagy (*Lamtor3, Snx20, Stx12*).

We next applied Ingenuity Pathway Analysis to genes with DMRs to identify enrichments of cellular pathways. This yielded 16 master regulators (Supplemental Table 7), of which 11 were inhibited in Off-HFD hearts (z-score<−2.0, *p*<0.05), including the transcriptional regulators Yin Yang 1 (*yy1*) and Mastermind Like Transcriptional Coactivator 1 (*maml1*); peptidases ADAM Metallopeptidase with Thrombospondin Type 1 Motif 5 (*adamts5*) and Plasminogen Activator, Tissue Type (*plat*); cell adhesion molecule Fat Atypical Cadherin 1 (*fat1*); and the Mitogen-activated Protein Kinase 4 (*mapk4*) family. Activated networks (z-score >2.0, *p*<0.05) included those involving cytokine colony-stimulating factor 1 (*csf1*), which regulates the production, differentiation, and function of macrophages; enzyme tumor necrosis factor (TNF)- alpha-induced protein 3 (*tnfaip3*); and the metalloproteinase inhibitor *timp3*.

We further sought to identify possible enrichment in motifs for transcription factors binding sites in correspondence of DMRs using the Hypergeometric Optimization of Motif EnRichment (HOMER) tool (41). This analysis yielded 14 transcription factor binding motifs specifically enriched in DMRs (Supplemental Table 8), including motifs recognized by GA Binding Protein Transcription Factor Subunit Alpha (GABPA), a factor involved in regulation of energy metabolism in the heart (50); ETS Proto-oncogene 1 (ETS1), which plays a crucial role in cardiac function (51); and ETS Variant Transcription Factor 2 (ETV2), a major regulator of cardiac and vascular development (52–54).

### Histological and metabolic changes in the heart of adult Off-HFD mice.

**Figure 6.**
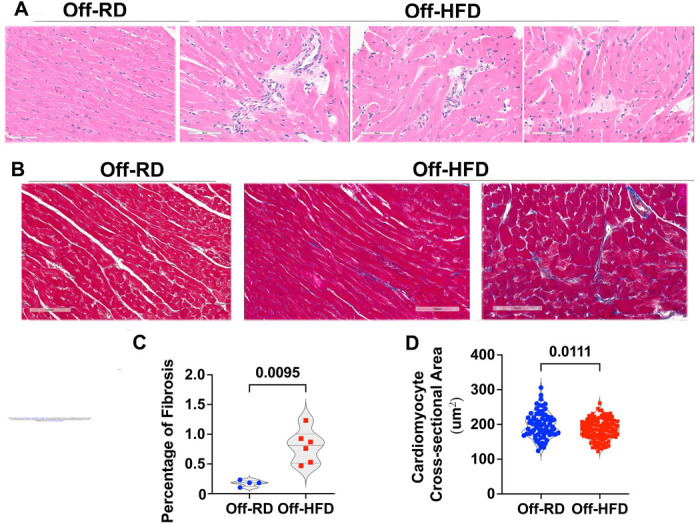
Histopathological evaluations. A, H&E staining of representative heart sections from Off-RD and Off-HFD mice showing mild perivascular inflammation in Off-HFD mice. Scale bar 60 μm. B, Masson’s trichrome staining of cardiac sections. Scale bars 100 μm (Off-RD), and 100 and 80 μm (Off-HFD). C, Quantification of cardiomyocyte cross-sectional area from trichrome-stained images. Overall, 135–150 cardiomyocytes were analyzed in each group of maternal diet. D, Quantification of fibrosis. Blue-stained areas in trichrome staining were color-thresholded using NIH ImageJ and quantitated. Data from males and females were pooled. Data are represented as individual values with lines at mean with SEM. N=3/sex/group of maternal diet, *p*-values are shown.

We next subjected Off-RD and Off-HFD hearts to routine histological analysis using H&E and trichrome-stained paraffin sections derived from four-month-old animals. Figure 6 illustrates some of the pertinent pathological differences between Off-RD and Off-HFD hearts. While Off-RD mice demonstrated normal tissue architecture, Off-HFD mice displayed perivascular inflammatory cell infiltrations potentially related to early ischemia (Fig.6A). We additionally sectioned paraffin-embedded Off-RD and Off-HFD hearts and applied trichrome staining to identify possible areas of fibrosis that contain collagen (Fig.6B). This revealed greater interstitial fibrosis in Off-HFD vs. Off-RD hearts (Fig.6B), specifically a four-fold increase in total area of fibrosis per mouse (*p*=0.0095, Fig.6C). Quantification of cardiomyocyte cross-sectional area in trichrome-stained sections further showed that despite the hypertrophy, Off-HFD hearts had slightly but significantly decreased cross-sectional area compared with Off-RD hearts (*p*=0.011, Fig.6D).

**Figure 7.**
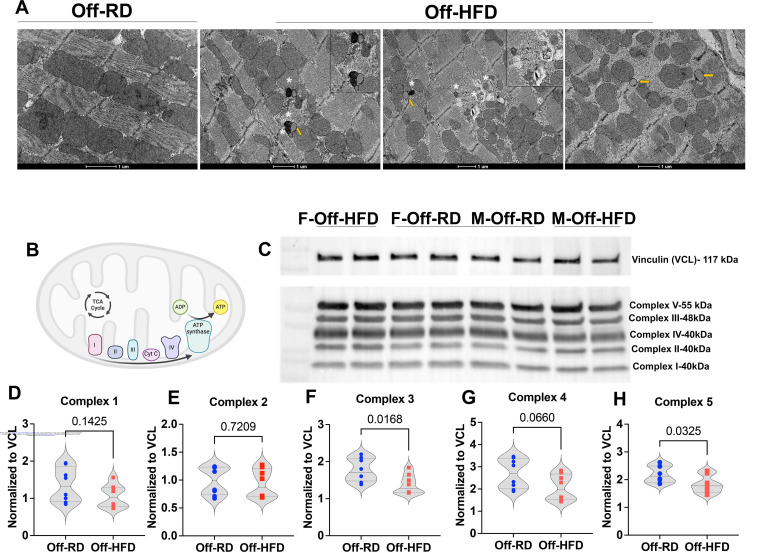
Ultrastructural and metabolic changes in the heart of Off-HFD mice. A, Transmission electron microscopy from 16-week-old mice showing dysregulations in mitochondrial alignment and increased accumulation of autolysosomes (asterisks) and lipid droplets (yellow arrows) in Off-HFD vs. Off-RD. Inserts present magnified views of autophagic vacuoles and lipid droplets. Scale bar: 1 mm. B-H, Western blots for expression of subunits of the mitochondrial electron transport chain, with Vinculin as loading control. B, Cartoon of the mitochondrial complexes, created with Biorender.com. C, Representative western blots for male and female Off-RD and Off-HFD. D-H, Quantification data for complex 1 (D), 2 (E), 3 (F), 4 (G), and 5 (H). Data were analyzed with two-way ANOVA followed by *t*-test. Data are represented as individual values with lines at mean with SEM. *P*-values are shown. Data from males and females were pooled. N=8/group of maternal diet.

We further assessed the ultrastructure of Off-RD and Off-HFD hearts using transmission electron microscopy. Although this evaluation did not show any gross alterations in cardiomyocytes, we found that the overall architecture of mitochondrial arrangement in relation to sarcomeres was significantly altered in Off-HFD hearts. While typically, mitochondria are densely packed in a well-organized array between sarcomeres, their transverse boundaries closely aligned with the Z-line, Off-HFD heart tissue showed bundles of mitochondria lacking this regular arrangement (Fig.7A). In addition, Off-HFD hearts presented accumulation of autophagic structures, including autophagosomes and autolysosomes (labeled with white asterisks), and increased number of lipid droplets (yellow arrows). To establish a link between irregular mitochondrial appearance and perturbed function, we performed western blots for protein subunits from five electron transport chain complexes (Fig. 7B–H). The results revealed significant decreases in expression of complexes 3 and 5 (Fig.7F and H), while no changes were observed for complexes 1, 2, and 4. As the electron microscopy images suggested accumulation of autophagic vacuoles in Off-HFD hearts (Fig.7A), we next measured the protein expression of regulators of autophagy at early (Transcription Factor EB, TFEB), mid (sequestosome p62), and late (Rubicon) stages of progression; however, no between-group differences were observed (Supplemental Figure 2).

### Immune profiles of adult Off-RD and Off-HFD hearts.

**Figure 8.**
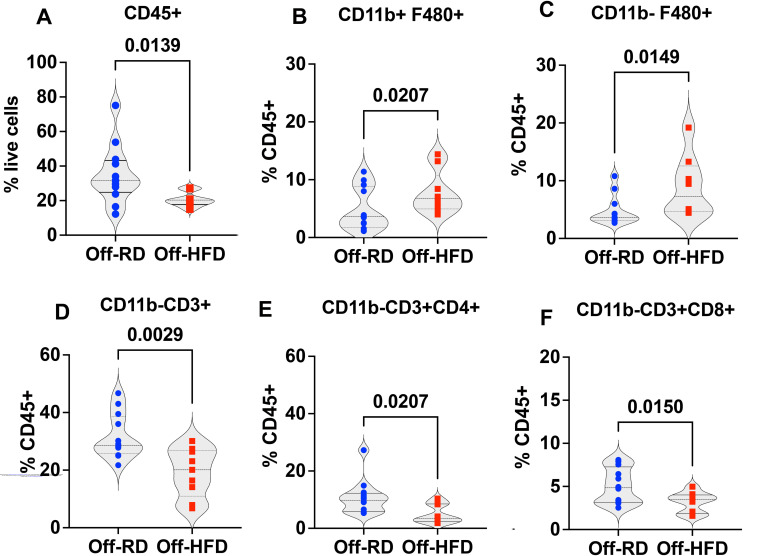
Flow cytometric analysis of immune cell populations in the heart of 16-week-old Off-RD and Off-HFD mice. Changes in heart immune complexity in Off-HFD as compared with Off-RD mice include: a decrease in CD45+ cells (A); increases in CD11b/F480 macrophages (B) and CD11b-/F480+ resident macrophages (C); and decrease in the percentage of CD3+ cells (D), including both CD4+ (E) and CD8+ T-cells (F). Data are represented as individual values with lines at mean with SEM. N=9–12/group of maternal diet, *p*-values are shown.

Inflammation has been recognized as a pathogenic feature of cardiac metabolic dysfunction and fibrosis (55–57). Accordingly, we conducted flow cytometry to examine immune cell populations in the hearts of four-month-old male and female Off-RD and Off-HFD mice. The gating strategy is illustrated in Supplemental Figure 3. Our data revealed a decrease in CD45^+^ cells (Fig.8A) alongside significant increases in CD11b^+^F480^+^ macrophages and CD11b^−^ F480^+^ resident macrophages in Off-HFD vs. Off-RD hearts (Fig.8B–C). In addition, T-cells were decreased in Off-HFD hearts, including CD3^+^, CD4^+^, and CD8^+^ cells (Fig.8D–F).

## DISCUSSION

While pharmacological interventions for obesity are advancing, the difficulty in reversing and maintaining weight loss due to relapse underscores the importance of prevention. Lifestyle choices are recognized as key determinants in the development of obesity and its comorbidities; however, findings from this study and others (16) highlight that intrauterine exposure to a maternal obesogenic diet also contributes to increased cardiovascular disease risk. Consequently, effective prevention strategies must start before birth and continue throughout the lifespan.

There is a substantial body of epidemiological data linking maternal obesity to cardiovascular dysfunction in offspring (16). Studies in children born to obese mothers have reported structural and functional cardiac dysregulations (58) including diastolic dysfunction, hypertrophy, and hypertension (58–61). Across animal models, intrauterine exposure to maternal obesity has also been shown to lead to cardiac hypertrophy in adult offspring, with structural and functional cardiac perturbations (62–64). In the present study, we utilized our mouse model of maternal diet-induced obesity to characterize how maternal environment affects offspring cardiac function. We focused our experiments on epigenetic, functional, metabolic, and immune aspects of the heart from offspring weaning to adult age. Consistent with epidemiological findings, our data revealed that despite being fed a regular diet only and living a stress-free life, offspring of HFD-fed mothers present dyslipidemia, cardiac hypertrophy, hypertension and perturbations in cardiac function in adult age. Importantly, our functional data show sexual dimorphism in cardiac adaptation to maternal obesity, with males being hypertensive and showing greater septal and posterior wall thickness in some (but not all) measurements, while females presented with reduced ejection fraction without any changes in LV diameter or volume. Similar increases in intraventricular septum thickness were previously reported in 14-, 20-, and 32-week-old human fetuses carried by obese mothers (65).

Notably, despite progressing cardiac hypertrophy, the cross-sectional areas of cardiomyocytes were reduced in Off-HFD mice, a surprising discovery that contrasts with previous reports (66). Reduced cross-sectional area could be explained by either remodeling of mitochondrial dysfunction or changes in signal transduction pathways such as p53, which has been shown to cause cardiomyocyte elongation without altering area (67). The mechanisms underlying this reduction are yet unclear, and experiments are underway to investigate this important phenomenon.

Intrauterine exposure to maternal obesity also has a demonstrated association with development of cardiac fibrosis (68–70). Interestingly, several groups have reported presence of cardiac fibrosis in animals that were placed on a high-fat diet feeding post-weaning (64). Our team has have previously reported cardiac fibrosis in non-human primate fetuses born to obese mothers (71). Data from this study also suggest accumulation of fibrotic tissue, although to lesser degree, in the hearts of adult Off-HFD mice even in the absence of superimposed dietary challenges.

Overall, our findings indicate an age-dependent progression of cardiac pathology in offspring of obese mothers. Maternal obesity clearly has long-lasting effects on offspring cardiac metabolism and contractile function via currently undefined mechanisms. While multiple mechanisms can detect alterations in contractility, we propose that a key component of this pathology is related to changes in mitochondrial respiration. Specifically, we found changes in cardiac energy metabolism, including reduced expression of subunits of complexes III and V of the mitochondrial electron transport chain, complexes that are crucial for cardiac development and function (72, 73). In addition, electron microscopy revealed alterations in mitochondrial positioning within the heart tissue ultrastructure. Previously, changes in cardiac mitochondrial structure and alignment and in activity of mitochondrial complexes have both been documented in a mouse model of maternal high-fat–high-sucrose diet (62). Importantly, the same study also revealed a transgenerational inheritance (both male- and female-transmitted) of cardiac mitochondrial defects in the descendants of obese females (62). Our electron microscopy data did not show any structural changes in cardiac mitochondria, which can be explained by differences in maternal diet (high-fat diet vs. high-fat–high-sucrose diet).

Mitochondrial dysfunction plays a crucial role in the programming effects of maternal obesity, potentially indicating metabolic inflexibility stemming from exposure to a dyslipidemic and inflamed intrauterine environment. We have previously reported that human neonates born to obese mothers exhibit systemic dyslipidemia that puts them at high risk of developing cardiovascular and metabolic diseases in later life (74). Here, despite being weaned to a regular diet only, adult Off-HFD mice also show development of dyslipidemia that includes greater levels of cholesterol, triglycerides, phospholipids, and non-esterified fatty acids. Our lab and others have previously demonstrated mitochondrial dysfunction and metabolic inflexibility in placentae of obese women (75) and in the hearts of sheep (76) and rats (77) born to obese mothers. Metabolic flexibility is clinically evaluated using indirect calorimetry by measuring the respiratory exchange ratio (RER), which represents the volume of carbon dioxide produced relative to the volume of oxygen consumed. Our findings revealed significantly lower RER values in adult Off-HFD mice, coupled with an impaired ability to transition between carbohydrate and lipid fuel sources during activity and rest, indicative of metabolic inflexibility. Similarly, low RER has previously been reported in obese individuals (78) and in patients with type 2 diabetes (79).

In addition to cardiac and metabolic features, pre-gravid obesity has been shown to modulate offspring immunity (80). While clinical studies are naturally limited in their investigative scope, several have profiled cord blood, shown to be an excellent representation of the fetal immune system at birth (81), and found changes in immune cell frequency and phenotype in the context of maternal obesity (82–85). Our previous reports have further identified accumulation of inflammation in the placentae of obese women, suggesting that maternal obesity exposes the fetus to chronic inflammation during development (86, 87). In our mouse model of maternal diet-induced obesity, a prior multiomics analysis of bone marrow and its myeloid cells in newly weaned Off-HFD and Off-RD mice uncovered significant metabolic and immune changes in the offspring of HFD-fed mothers (29, 31). This indicates that immune and immunometabolic disruptions in the offspring of obese mothers occur early, before the manifestation of symptomatic metabolic disease. This study revealed significant alterations in immune cell populations in adult Off-HFD hearts. More specifically, although total immune cell numbers were reduced, there were increases in resident cardiac macrophages and infiltrated macrophages, immune cells crucial for adaptive cardiac remodeling (88).

Expansion of resident macrophages has been already reported in response to immune challenge, and was found to be associated with excessive production of collagen and accumulation of cardiac fibrosis (89). T lymphocytes also play important roles in cardiac homeostasis and response to injury (90). Subsets of CD4+ nonspecific effector T cells have demonstrated the capability to protect against post-inflammatory fibrosis in an experimental model of myocarditis (91), and it has been suggested that cardiac CD8+T-cells regulate the conversion of both cardiac-resident macrophages and infiltrated macrophages into cardioprotective macrophages, which are essential for myocardial adaptive responses. Here, we observed reductions in both CD4+ and CD8+ T-cell subsets; however, the roles of these changes in the observed cardiac dysfunction remains unclear. Further refinement of cardiac macrophage and T-cell subtypes, along with a more comprehensive understanding of their roles in the response to an adverse maternal environment, remains crucial. Our laboratory is currently pursuing this line of investigation.

The above findings indicate that offspring cardiac adaptation to maternal obesity is underscored by metabolic, immune, and functional changes, prompting questions of when and how the maternal environment affects fetal hearts. Alterations in epigenetic modifications are key in regulating the functioning and expression of genes related to cardiovascular disease through various mechanisms such as DNA methylation, histone modification, and noncoding RNA regulation (92). Importantly, epigenetic changes have previously been described in the setting of maternal obesity (93, 94). A clinical study that included 40 mother-infant dyads revealed strong association of maternal blood metabolites with fetal umbilical cord blood DNA methylation patterns (95), and exposure to maternal obesity during development is associated with changes in global DNA methylation of CpG sites and islands in the offspring’s white adipose tissue, specifically in genes involved in adipogenesis (96, 97). These findings suggest that epigenetic modifications may provide a mechanism for programming of obesity and dyslipidemia in the offspring of obese mothers. We and others have previously reported dysregulations of fetal cardiac miRNAs in a non-human primate model of maternal obesity (71, 98). In this study, we profiled the methylation status of cardiac genes in the hearts of newly weaned three-week-old mice with the aim of identifying sets of cardiac genes that are differentially methylated early in life, prior to the appearance of obesity and metabolic perturbations. The results revealed significant changes in DNA methylation for Off-HFD mice, with enrichment of genes responsible for cardiac development, cardiac hypertrophy, metabolism, and immune responses and inflammation. Further experiments are needed to determine 1) what is the contribution of differentially methylated genes and pathways to the functional, immune, and metabolic dysregulations seen in the offspring of obese mothers; and 2) does the differential cardiac methylation pattern in young Off-HFD persist into adult life and into future generations?

Collectively, our data revealed metabolic, functional, and immune perturbations in the hearts of offspring of obese mothers. We also identified early-life cardiac epigenetic modifications that precede later-life cardiac abnormalities. While the various observed alterations may not be immediately catastrophic, they likely enhance the susceptibility of the offspring to secondary nutritional, psychological, or environmental insults, and increase the risk of future cardiovascular disease. Heart disease remains the leading cause of death in the U.S., and many of the contributing risk factors, including high blood pressure and obesity, continue to become more widespread at alarming rates (2). Undoubtedly, unhealthy dietary and lifestyle choices increase a person’s risk of developing cardiovascular disease. However, the present work suggest that exposure to maternal obesity *per se* predisposes the offspring to heart disease independent of their lifestyle choices. Understanding the mechanisms that underlie this predisposition could facilitate the prevention of cardiac dysfunction in future offspring of mothers with obesity, for example through targeted manipulation of immune cells or of mitochondrial function to reduce fibrosis and improve heart energy metabolism and contractility, areas that represent promising fields of active study.

## Supplementary Material

Supplement 1

Supplement 2

Supplement 3

Supplement 4

Supplement 5

Supplement 6

Supplement 7

Supplement 8

Supplement 9

Supplement 10

Supplement 11

## Data Availability

DNA methylation data were submitted to Gene Expression Omnibus (GEO) repository, the accession number is GSE294642.
