# Supplementary figures and images for: Maternal obesity programs cardiac remodeling in offspring via epigenetic, metabolic, and immune dysregulations
Source: bioRxiv. 2025 May 27:2025.04.15.648971. Preprint. [Version 2] doi: 10.1101/2025.04.15.648971 (PMC12154923; doi:10.1101/2025.04.15.648971)

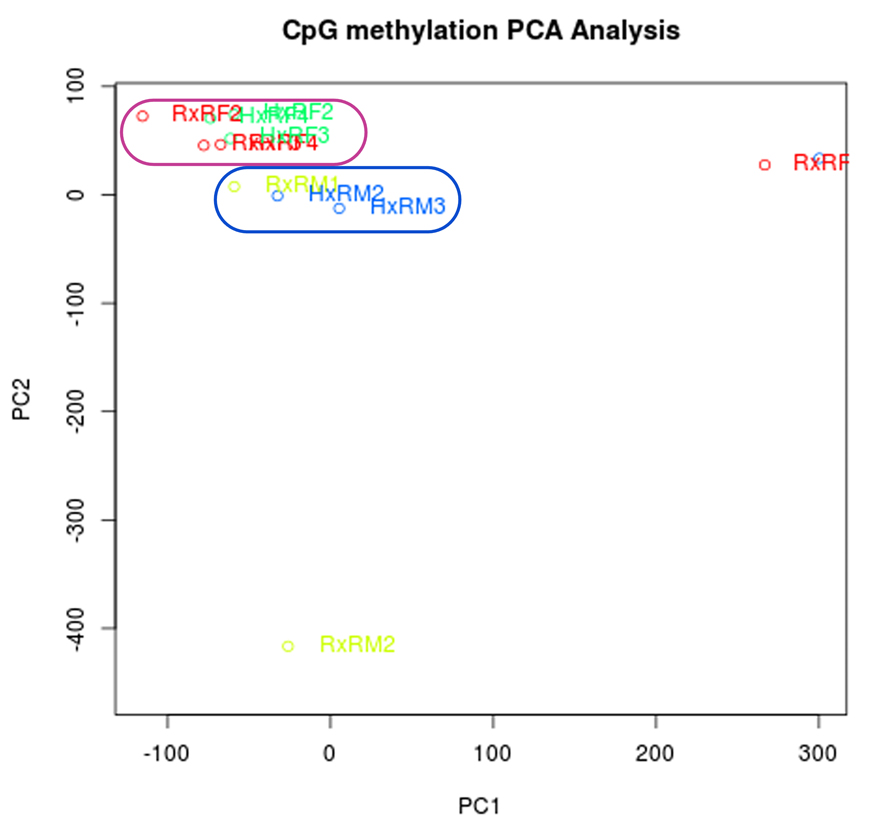

Supplement: Supplement 1 [file media-1.jpg]

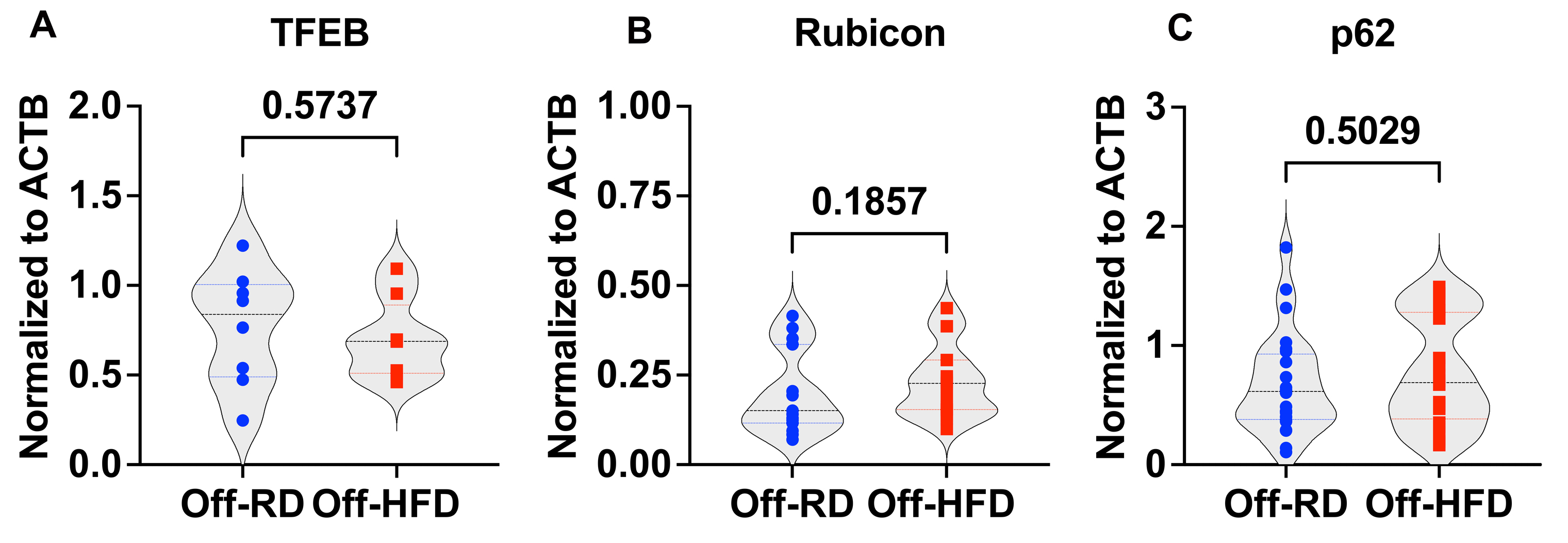

Supplement: Supplement 2 [file media-2.jpg]

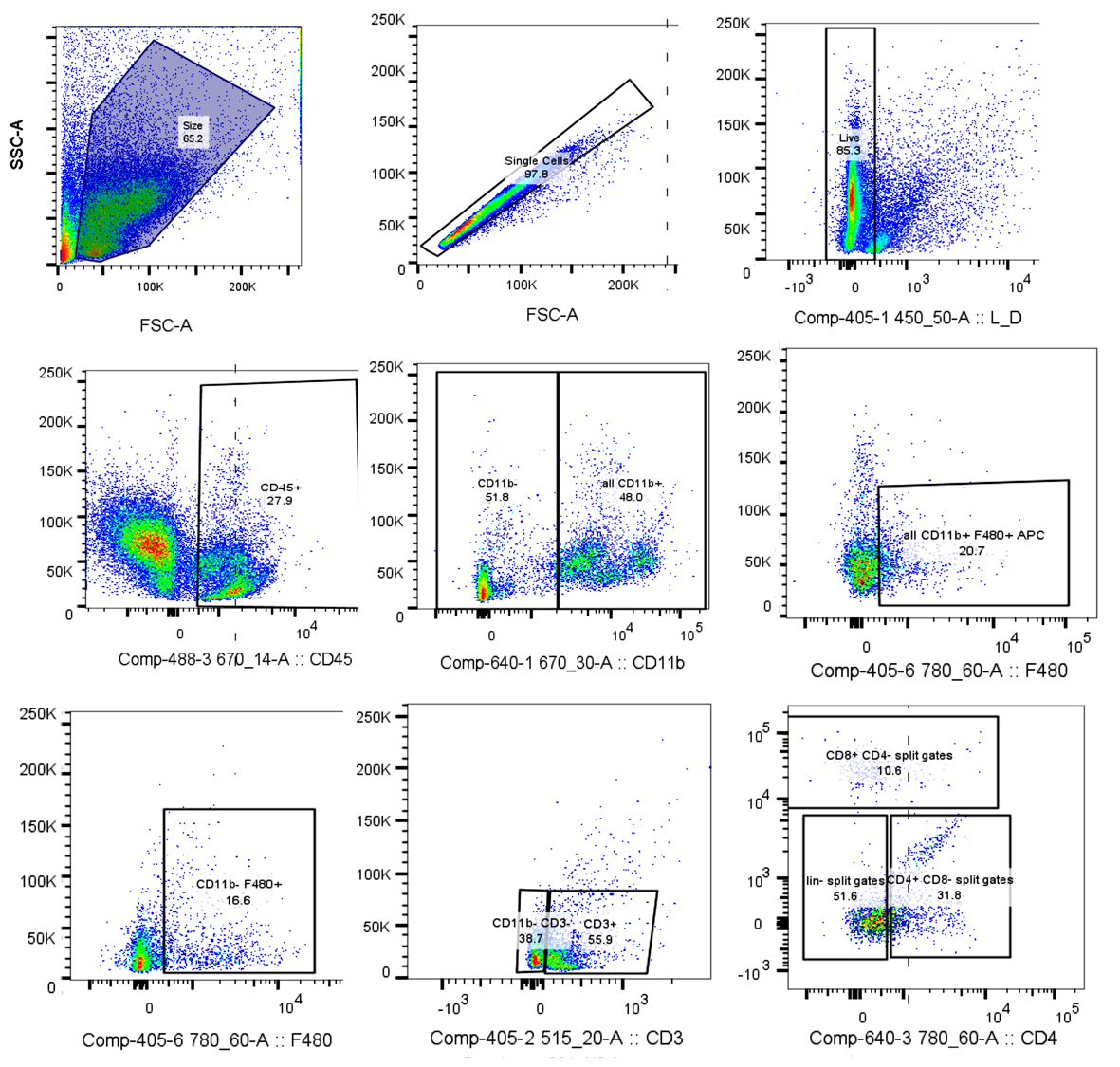

Supplement: Supplement 11 [file media-11.jpg]
