# Supplementary material for: Maternal obesity programs cardiac remodeling in offspring via epigenetic, metabolic, and immune dysregulations
Source: bioRxiv. 2025 May 27:2025.04.15.648971. Preprint. [Version 2] doi: 10.1101/2025.04.15.648971 (PMC12154923; doi:10.1101/2025.04.15.648971)
Supplement: Supplement 3 [file media-3.docx]

**Supplemental Table 1**. Flow cytometry antibody panel.

| Target | Fluorochrome | Clone | Company | Cat # |
| --- | --- | --- | --- | --- |
| CD45 | BUV395 Rat Anti-Mouse | 30-F11 | BD Biosciences | 564279 |
| CD4+ | BUV615 Rat Anti-Mouse | RM4-5 | BD Biosciences | 751486 |
| CD19+ | BUV661 Rat Anti-Mouse | 1D3 | BD Biosciences | 612971 |
| CD3 Molecular Complex | BUV737 Rat Anti-Mouse CD3 | 17A2 | BD Biosciences | 612803 |
| CD8+ | BUV805 Rat Anti-Mouse CD8a | 53-6.7 | BD Biosciences | 612898 |
| MHCII | eFluor™ 450 | M5/114.15.2 | Invitrogen | 48-5321-82 |
| CD11c | Brilliant Violet 605™ anti-mouse | N418 | BioLegend | 117334 |
| CD86 | Brilliant Violet 650™ anti-mouse | GL-1 | BioLegend | 105035 |
| F4/80 | Brilliant Violet 785™ anti-mouse F4/80 Antibody | BM8 | BioLegend | 123141 |
| Ly-6C | PerCP/Cyanine5.5 anti-mouse | HK1.4 | BioLegend | 128012 |
| CD11b | Alexa Fluor® 700 anti-mouse/human | M1/70 | BioLegend | 101222 |
