# Supplementary material for: Maternal obesity programs cardiac remodeling in offspring via epigenetic, metabolic, and immune dysregulations
Source: bioRxiv. 2025 May 27:2025.04.15.648971. Preprint. [Version 2] doi: 10.1101/2025.04.15.648971 (PMC12154923; doi:10.1101/2025.04.15.648971)
Supplement: Supplement 4 [file media-4.docx]

**Supplemental Table 2**. Echocardiographic parameters 4-month-old Off-RD and Off-HFD mice. Data are reported as mean±SEM for 5-7 mice/sex/experimental group. *, p < 0.05 in Off-HFD vs. Off-RD within each sex, ^#^, p<0.05 in males vs. females within each group of maternal diet.

| **Parameter** | **Males** | | **Females** | |
| --- | --- | --- | --- | --- |
|  | **Off-RD** | **Off-HFD** | **Off-RD** | **Off-HFD** |
| Body weight (g) | 30.24±1.13 | 32.07±0.69 | 26.56±0.53^#^ | **34.14±1.47*** |
| Left ventricular outflow tract (mm) | 1.37±0.08 | 1.250±0.04 | 1.66±0.05 | 1.20±0.05 |
| Left ventricular outflow tract cardiac output (mm/min) | 42.27±9.44 | 36.83±5.25 | 26.07±4.03 | 25.27±1.07 |
| Left ventricular outflow tract stroke volume (ul) | 101.85±18.39 | 75.54±10.02 | 60.06±8.11 | 66.55±2.59 |
| Heart Rate (bpm) | 402.79±36.14 | 496.98±59.56 | 453.75±69.81 | 380.71±15.42 |
| Intra-ventricular septum  thickness, diastole (mm) | 0.61±0.02 | **0.77±0.03*** | 0.75±0.06 | 0.68±0.03 |
| Intra-ventricular septum  thickness, systole (mm) | 0.70±0.05 | 0.79.04 | 0.87±0.04^#^ | 0.75±0.08 |
| Left ventricular inner  diameter, diastole (mm) | 4.40±0.09 | 4.44.21 | 3.97±0.11^#^ | 3.96±0.06 |
| Left ventricular inner  diameter, systole (mm) | 3.55±0.09 | 3.53±0.19 | 2.95±0.09^#^ | 3.09±0.14 |
| Left ventricular posterior  wall thickness, diastole (mm) | 0.71±0.04 | 0.77±0.06 | 0.74±0.05 | **0.79±0.05*** |
| Left ventricular posterior  wall thickness, systole (mm) | 0.62±0.03 | **0.83±0.05*** | 0.86±0.06 | 0.80±0.08 |
| VTI (mm) | 66.03±4.61 | 60.25±4.23 | 54.94±4.68 | 60.05±5.91 |
| LVOT VTI, mean velocity (mm/s) | 971.20±55.44 | 893.30±61.27 | 694.97±32.84^#^ | 791.96±61.85 |
| LVOT VTI, mean gradient (mmHg) | 3.82±0.44 | 3.31±0.44 | 1.95±0.18^#^ | 2.57±0.39 |
| LVOT VTI, peak velocity (mm/s) | 1586.18±78.60 | 1453.63±87.48 | 1186.77±57.25^#^ | 1265.81±89.96^#^ |
| LVOT VTI, peak gradient (mmHg) | 10.16±1.015 | 8.70±1.02 | 5.68±0.52^#^ | 6.54±0.91^#^ |
| LVOT, B-mode, length (mm) | 1.37±0.08 | 1.25±0.04 | 1.16±0.05 | 1.20±0.05 |
| Ejection fraction (%) | 40.02±3.21 | 43.13±3.93 | 56.49±1.69^#^ | **43.92±4.02*** |
| Fractional shortening (%) | 19.45±1.8 | 21.49±2.33 | 29.64±1.65^#^ | **22.00±2.32*** |
| Left Ventricular Mass (mg) | 110.12±9.30 | 137.46±10.90 | 106.62±8.54 | 103.99±6.17^#^ |
| Left Ventricular Mass (Corrected) (mg) | 88.10±7.44 | 109.97±8.72 | 85.29±6.83 | 83.19±4.93^#^ |
| Left Ventricular Volume, diastole (ul) | 88.23±4.61 | 94.09±8.70 | 69.49±4.45^#^ | 68.33±2.74^#^ |
| Left Ventricular Volume, systole (ul) | 52.73±3.20 | 53.92±6.78 | 34.07±2.55^#^ | 38.19±4.62 |
