# Supplementary material for: Maternal obesity programs cardiac remodeling in offspring via epigenetic, metabolic, and immune dysregulations
Source: bioRxiv. 2025 May 27:2025.04.15.648971. Preprint. [Version 2] doi: 10.1101/2025.04.15.648971 (PMC12154923; doi:10.1101/2025.04.15.648971)
Supplement: Supplement 5 [file media-5.docx]

**Supplemental Table 3.** Number of reads per sample before and after trimming. Abbreviations: F, females, M, males.

| **Sample** | **Input Reads** | **Quality Truncated Sequences** | **Sequences Removed due to Length** | **RRBS Reads Trimmed 2bp** |
| --- | --- | --- | --- | --- |
| F1 Off-HFD | 14212431 | 2352082 (16.5%) | 517606 (3.6%) | 4145610 (29.2%) |
| F2 Off-HFD | 17690547 | 3867737 (21.9%) | 3674297 (20.8%) | 4332203 (24.5%) |
| F3 Off-HFD | 18425556 | 3144272 (17.1%) | 3835455 (20.8%) | 4449561 (24.1%) |
| F4 Off-HFD | 19205539 | 4068972 (21.2%) | 6128006 (31.9%) | 4045707 (21.1%) |
| M1 Off-HFD | 8283 | 3659 (44.2%) | 3823 (46.2%) | 1539 (18.6%) |
| M2 Off-HFD | 17530080 | 3648490 (20.8%) | 5048042 (28.8%) | 3734441 (21.3%) |
| M3 Off-HFD | 20595634 | 7271594 (35.3%) | 7441955 (36.1%) | 4057275 (19.7%) |
| M4 Off-HFD | 18028380 | 3901249 (21.6%) | 5637435 (31.3%) | 4023292 (22.3%) |
| F1 Off-RD | 16823113 | 2112459 (12.6%) | 691350 (4.1%) | 5059643 (30.1%) |
| F2 Off-RD | 16700039 | 2205176 (13.2%) | 557934 (3.3%) | 5129896 (30.7%) |
| F3 Off-RD | 17398685 | 2122524 (12.2%) | 735437 (4.2%) | 5146266 (29.6%) |
| F4 Off-RD | 17194403 | 2880341 (16.8%) | 2811782 (16.4%) | 4376713 (25.5%) |
| M1 Off-RD | 18819759 | 2421443 (12.9%) | 1878038 (10.0%) | 5309345 (28.2%) |
| M2 Off-RD | 16617492 | 2031450 (12.2%) | 1110523 (6.7%) | 4930790 (29.7%) |
| M3 Off-RD | 21559809 | 3050234 (14.1%) | 7120305 (33.0%) | 4478794 (20.8%) |
| M4 Off-RD | 14731018 | 2419171 (16.4%) | 1638980 (11.1%) | 4085200 (27.7%) |
