# Supplementary material for: Maternal obesity programs cardiac remodeling in offspring via epigenetic, metabolic, and immune dysregulations
Source: bioRxiv. 2025 May 27:2025.04.15.648971. Preprint. [Version 2] doi: 10.1101/2025.04.15.648971 (PMC12154923; doi:10.1101/2025.04.15.648971)
Supplement: Supplement 6 [file media-6.docx]

**Supplemental Table 4**. Hypomethylated DMRs with Sidak p-value<0.05 and percentage of methylation change between Off-RD and Off-HFD >10%.

| Gene | chr | Length | % methyl  change | Sidak_Pval | Avg methyl  Off-HFD | Avg methyl  Off-RD | Gene description |
| --- | --- | --- | --- | --- | --- | --- | --- |
| H13 | 2 | 180 | -43.65 | 7.36E-10 | 41.71 | 85.37 | Minor histocompatibility antigen H13 |
| Impact | 18 | 288 | -42.68 | 6.33E-17 | 40.12 | 82.81 | Impact, RWD domain protein (Impact) |
| Snx20 | 8 | 13 | -42.13 | 0.02402 | 9.98 | 52.12 | Sorting nexin 20 |
| Lamtor3 | 3 | 75 | -28.36 | 0.004995 | 29.12 | 57.48 | Late endosomal/lysosomal adaptor, MAPK and MTOR activator 3 |
| Mindy4 | 6 | 71 | -27.28 | 9.49E-07 | 26.45 | 53.74 | Probable ubiquitin carboxyl-terminal hydrolase MINDY-4 |
| Pecam1 | 11 | 74 | -26.98 | 0.0004263 | 19.35 | 46.34 | Platelet/endothelial cell adhesion molecule 1 |
| Kcnb1 | 2 | 18 | -26.81 | 0.02293 | 20.90 | 47.72 | Potassium voltage-gated channel subfamily B member 1 |
| Dock9 | 14 | 74 | -24.12 | 0.0001946 | 31.11 | 55.24 | Dedicator of cytokinesis protein 9 isoform 5 |
| Elk3 | 10 | 21 | -22.95 | 0.0007803 | 36.53 | 59.49 | ETS domain-containing protein Elk-3 |
| Eng | 2 | 170 | -22.49 | 0.009755 | 24.03 | 46.52 | Endoglin |
