# Supplementary material for: Maternal obesity programs cardiac remodeling in offspring via epigenetic, metabolic, and immune dysregulations
Source: bioRxiv. 2025 May 27:2025.04.15.648971. Preprint. [Version 2] doi: 10.1101/2025.04.15.648971 (PMC12154923; doi:10.1101/2025.04.15.648971)
Supplement: Supplement 7 [file media-7.docx]

**Supplemental Table 5.** Hypermethylated DMRs with Sidak p-value<0.05 and percentage of methylation change between Off-RD and Off-HFD >10%.

| Gene | Chr | Length | % methyl change | Sidak_Pval | Avg methyl Off-HFD | Avg methyl Off-RD | Gene description |
| --- | --- | --- | --- | --- | --- | --- | --- |
| Twf1 | 15 | 75 | 25.10 | 0.002114 | 42.73 | 17.62 | Twinfilin actin binding protein 1 |
| Golim4 | 3 | 74 | 24.46 | 0.0003081 | 26.26 | 1.80 | Golgi integral membrane protein 4 |
| Atp6v1g1 | 4 | 50 | 23.18 | 3.63E-06 | 25.59 | 2.41 | ATPase, H+ transporting, lysosomal V1 subunit G1 |
| Notch1 | 2 | 74 | 17.58 | 0.008981 | 18.34 | 0.76 | Neurogenic locus notch homolog protein 1 |
| Adamts1 | 16 | 74 | 12.23 | 0.03939 | 13.40 | 1.16 | A disintegrin and metalloproteinase with thrombospondin motifs 1 |
| Gle1 | 2 | 68 | 8.89 | 0.0153 | 9.94 | 1.04 | Nucleoporin GLE1 |
| Cebpa | 7 | 116 | 7.82 | 8.72E-06 | 9.65 | 1.82 | CCAAT/enhancer-binding protein alpha |
| Mzf1 | 7 | 127 | 7.45 | 0.005353 | 9.46 | 2.01 | Myeloid zinc finger 1 |
| C2cd4b | 9 | 54 | 6.98 | 0.01007 | 9.67 | 2.69 | C2 calcium-dependent domain containing 4B |
| Cadm1 | 9 | 203 | 5.21 | 0.001791 | 5.46 | 0.24 | Cell adhesion molecule 1 |
