# Supplementary material for: Maternal obesity programs cardiac remodeling in offspring via epigenetic, metabolic, and immune dysregulations
Source: bioRxiv. 2025 May 27:2025.04.15.648971. Preprint. [Version 2] doi: 10.1101/2025.04.15.648971 (PMC12154923; doi:10.1101/2025.04.15.648971)
Supplement: Supplement 8 [file media-8.docx]

**Supplemental Table 6**. DMRs and related genes with previously shown role in cardiovascular function.

| Gene | Identifier | Category | | Functional Annotation |
| --- | --- | --- | --- | --- |
| Adamts1 | 10090.ENSMUSP00000023610 | GO Process | Developmental process involved in reproduction, heart morphogenesis, cardiac chamber development | |
| Adgrf5 | 10090.ENSMUSP00000109229 | GO Process | Cardiac hypertrophy, vasculature development, myeloid cell homeostasis, regulation of immune response, lipid metabolism | |
| Atp6v1g1 | 10090.ENSMUSP00000048770 | GO Process | mTOR signaling, Cellular response to increased oxygen levels, ATPase binding, lysosomal formation, innate immune system | |
| C2cd4b | 10090.ENSMUSP00000131138 | GO Process | Inflammatory response, lipid metabolism, regulation of fatty acids levels | |
| Cebpa | 10090.ENSMUSP00000096129 | GO Process | Organism development, cardiovascular system phenotype, immune response, macrophage differentiation, lipid metabolism, mitochondrial function | |
| Chml | 10090.ENSMUSP00000100600 | GO Process | Nitrogen compound metabolic process. Regulation of cell metabolism, vesicle-mediated transport, Abnormal postnatal growth/weight/body size | |
| Commd1 | 10090.ENSMUSP00000124719 | GO Process | Regulation of sodium ion transport, vesicle-mediated transport, response to stress, regulation of cell communication | |
| Cradd | 10090.ENSMUSP00000050295 | GO Process | Cell cycle checkpoint signaling, mitotic cell cycle, DNA damage response, cell death | |
| Cry1 | 10090.ENSMUSP00000020227 | GO Process | Carbohydrate metabolic process, protein phosphorylation, gluconeogenesis, mitochondrial function, circadian behavior | |
| Elk3 | 10090.ENSMUSP00000008542 | GO Process | Angiogenesis, regulation of transcription, metabolic process. | |
| Eng | 10090.ENSMUSP00000009705 | GO Process | Angiogenesis, EMT, protein phosphorylation, heart looping, septum morphogenesis, cardiac chamber development, cardiac ventricle morphogenesis | |
| Epas1 | 10090.ENSMUSP00000024954 | GO Process | Angiogenesis, response to hypoxia, regulation of heart rate, myeloid cell homeostasis, immune response, mitochondrial function. | |
| Esam | 10090.ENSMUSP00000002011 | GO Process | Cell-cell junction assembly, cell adhesion, actin polymerization, Regulation of actin cytoskeleton reorganization | |
| F2r | 10090.ENSMUSP00000061754 | GO Process | Leukocyte homeostasis, cytokine production, immune system development, Abnormal cardiovascular development, cardiac hypertrophy | |
| H13 | 10090.ENSMUSP00000086460 | GO Process | In utero embryonic development, proteolysis, metabolism, response to stress, intracellular protein transport, connective tissue, abnormal heart morphology, aging | |
| Impact | 10090.ENSMUSP00000025290 | GO Process | Negative regulation of transcription by RNA polymerase II, response to oxidative stress, cellular response to starvation. | |
| Ino80c | 10090.ENSMUSP00000114643 | GO Process | Telomere maintenance, regulation of DNA replication, response to stress | |
| Jmjd6 | 10090.ENSMUSP00000047570 | GO Process | Protein demethylation, phagocytosis, heart development | |
| Kcnb1 | 10090.ENSMUSP00000147093 | GO Process | Cellular glucose homeostasis, action potential, potassium ion transport, GLP-1 signaling, regulation of insulin secretion | |
| Lamtor3 | 10090.ENSMUSP00000130811 | GO Process | Signal transduction, cell metabolism, mTOR signaling, MAPK cascade, lysosome, late endosome, autophagy | |
| Nfkbil1 | 10090.ENSMUSP00000035452 | GO Process | Regulation of cytokine production, metabolic dysfunction, I-kappaB kinase/NF-kappaB signaling, immune response, signal transduction | |
| Notch1 | 10090.ENSMUSP00000028288 | GO Process | Adaptive immune response based on somatic recombination of immune receptors built from immunoglobulin superfamily domains, endocardium, conduction system, valve development | |
| Nr3c2 | 10090.ENSMUSP00000105539 | GO Process | Regulation of systemic arterial blood pressure by hormone, G protein-coupled receptor signaling pathway | |
| Nrap | 10090.ENSMUSP00000073228 | GO Process | Actin filament organization, heart development, actomyosin structure organization, cardiomyocyte differentiation | |
| Nrp2 | 10090.ENSMUSP00000109794 | GO Process | Heart development | |
| Pecam1 | 10090.ENSMUSP00000102408 | GO Process | Cell morphogenesis, angiogenesis, endothelium development. Immune system process, protein phosphorylation. | |
| Plagl1 | 10090.ENSMUSP00000112847 | GO Process | Regulation of transcription, DNA-templated, maternal and paternal imprinting, Decreased birth weight, abnormal heart size | |
| Plek2 | 10090.ENSMUSP00000021544 | GO Process | Cytoskeleton organization, Actin cytoskeleton organization | |
| Ppp1r16b | 10090.ENSMUSP00000062615 | GO Process | Endothelial cell development, Regulation of phosphatidylinositol 3-kinase signaling, regulation of cell proliferation | |
| Set | 10090.ENSMUSP00000099930 | GO Process | Chromatin organization and remodeling, cell death, cell metabolism | |
| Shb | 10090.ENSMUSP00000060433 | GO Process | Angiogenesis, Immune response-activating cell surface receptor signaling pathway, Lymphocyte activation and proliferation | |
| Snx20 | 10090.ENSMUSP00000034087 | GO Process | protein transport, lipid binding, autophagy, lysosome | |
| Stard3 | 10090.ENSMUSP00000018311 | GO Process | Lipid metabolism , Steroid biosynthetic process, lipid and cholesterol transport, Vesicle-mediated transport | |
| Stx12 | 10090.ENSMUSP00000030698 | GO Process | Autophagosome assembly, lipid transport, autophagy, vesicle-mediated transport | |
| Twf1 | 10090.ENSMUSP00000023087 | GO Process | Regulation of protein phosphorylation, cytoskeleton organization, cardiac hypertrophy, cell metabolism | |
| Ubb | 10090.ENSMUSP00000019649 | GO Process | Cell morphogenesis, microtubule-based process, protein ubiquitination and degradation, cellular senescence | |
