# Supplementary material for: Maternal obesity programs cardiac remodeling in offspring via epigenetic, metabolic, and immune dysregulations
Source: bioRxiv. 2025 May 27:2025.04.15.648971. Preprint. [Version 2] doi: 10.1101/2025.04.15.648971 (PMC12154923; doi:10.1101/2025.04.15.648971)
Supplement: Supplement 9 [file media-9.docx]

**Supplemental Table 7.** Master regulators identified by Ingenuity Pathway Analysis (IPA) within genes with DMRs.

| Master Regulator | Type of Molecule | Status | Z-score | -log(p-value) | Participating regulators |
| --- | --- | --- | --- | --- | --- |
| YY1 | transcription regulator | Inhibited | -2.87 | 5.49 | AP2 (family), CDKN1B, CEBPA, CEBPB, CREB1, EIF4E, ERK1/2 (family), ESTROGEN RECEPTOR |
| ADAMTS5 | peptidase | Inhibited | -2.84 | 3.90 | AKT (family), EGFR, ERK1/2(family), NFKB |
| PRKD1 | kinase | Inhibited | -2.65 | 6.11 | AKT (family), AR, CREB1, EPAS1, ERK1/2 (family) |
| XRCC5 | enzyme | Inhibited | -2.47 | 5.24 | AHR, AKT (family), AR, CEBPB, CHEK1, CREB1, CREBBP, ESR1 |
| ABRAXAS2 | ubiquitination protein | Inhibited | -2.33 | 6.69 | AHR, AKT (family), Casp1, ERBB2, ERK1/2 (family), IFNGR1, IRF3, IRF8, JAK2 |
| MAP4K (family) | group | Inhibited | -2.13 | 4.01 | ESR1, IRF3, JUN, MAP2K1 |
| FAT1 | cell adhesion protein | Inhibited | -2.12 | 2.68 | JUN, SAC, STAT5NB (family), WWTR1, YAP1 |
| SAPK (family) | group | Inhibited | -2.12 | 2.75 | JUN, JUNB, MAPK14, MAPK9 |
| MAML1 | transcription regulator | Inhibited | -2.12 | 4.14 | AKT1, CEBPB, CREB1, CREBBP, EP300, ERBB2, ESTROGEN RECEPTOR (family), JUN, LEF1 |
| PDLIM2 | actin-regulating protein | Inhibited | -2.06 | 4.52 | AP1 (family), CAV1, CREBBP, EGR2, FOS, GSDMD, IFNG, IL12, IL1B |
| PLAT | peptidase | Inhibited | -2.00 | 4.06 | ERK1/2 (family), FOS, IRF3, JUN, MTOR, NFKB (complex), P38 MAPK (family) |
| CSF1 | cytokine | Activated | 2.00 | 2.30 | CSF1 |
| RUNX1 | transcription regulator | Activated | 2.00 | 2.70 | RUNX1 |
| TNFAIP3 | enzyme | Activated | 2.00 | 4.21 | ERK1/2 (family), IL18, IRF3, JUN, MAP2K1, MYD88, NFKB (complex), NFKBIA, RELA |
| TIMP3 | inhibitor of matrix metalloproteinase | Activated | 3.21 | 3.56 | ADAM10, ADAM12, ADAM17,AKT (family), EGFR, ERK1/2 (family), KOR, NFKB (complex), RAF1, RELA |
