# Supplementary material for: Maternal obesity programs cardiac remodeling in offspring via epigenetic, metabolic, and immune dysregulations
Source: bioRxiv. 2025 May 27:2025.04.15.648971. Preprint. [Version 2] doi: 10.1101/2025.04.15.648971 (PMC12154923; doi:10.1101/2025.04.15.648971)
Supplement: Supplement 10 [file media-10.docx]

**Supplemental Table 8**. Enrichment of transcription factor binding sites within significant DMRs identified using HOMER Motif Enrichment software.

| **Rank** | **Motif** | **Name** | **P-value** | **q-value (Benjamini)** | **% of Targets Sequences with Motif** |
| --- | --- | --- | --- | --- | --- |
| 1 | 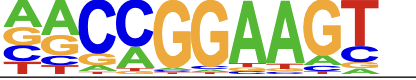 | GABPA(ETS)/Jurkat-GABPa-ChIP-Seq(GSE17954)/Homer | 1.00E-06 | 0.0001 | 70.42% |
| 2 | 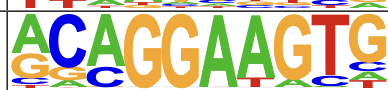 | ETS1(ETS)/Jurkat-ETS1-ChIP- Seq(GSE17954)/Homer | 1.00E-06 | 0.0001 | 73.24% |
| 3 | 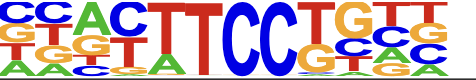 | Etv2(ETS)/ES-ER71-ChIP-Seq(GSE59402)/Homer | 1.00E-06 | 0.0001 | 67.61% |
| 4 | 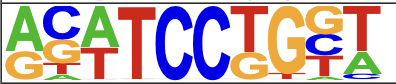 | SPDEF(ETS)/VCaP-SPDEF-ChIP-Seq(SRA014231)/Homer | 1.00E-06 | 0.0002 | 70.42% |
| 5 | 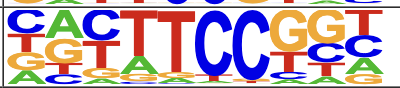 | Fli1(ETS)/CD8-FLI-ChIP-Seq(GSE20898)/Homer | 1.00E-05 | 0.0002 | 77.46% |
| 6 | 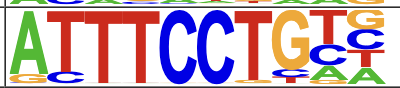 | EWS:ERGfusion(ETS)/CADO_ES1-EWS:ERG-ChIP-Seq(SRA014231)/Homer | 1.00E-04 | 0.0018 | 50.70% |
| 7 | 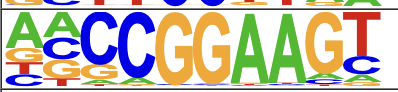 | ELF1(ETS)/Jurkat-ELF1-ChIP-Seq(SRA014231)/Homer | 1.00E-04 | 0.0059 | 50.70% |
| 8 | 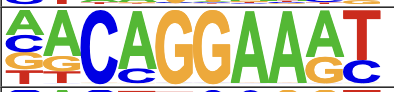 | EWS:FLI1fusion(ETS)/SK_N_MC-EWS:FLI1-ChIP-Seq(SRA014231)/Homer | 1.00E-04 | 0.0082 | 46.48% |
| 9 | 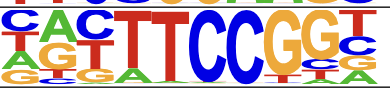 | Elk4(ETS)/Hela-Elk4-ChIP-Seq(GSE31477)/Homer | 1.00E-04 | 0.0088 | 54.93% |
| 10 | 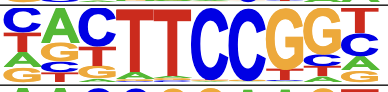 | Elk1(ETS)/Hela-Elk1-ChIP-Seq(GSE31477)/Homer | 1.00E-03 | 0.0175 | 53.52% |
| 11 | 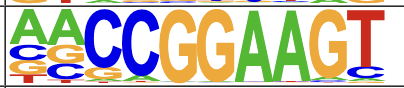 | ETS(ETS)/Promoter/Homer | 1.00E-03 | 0.0176 | 36.62% |
| 12 | 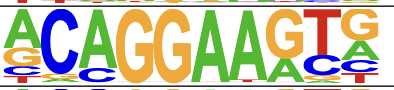 | ERG(ETS)/VCaP-ERG-ChIP-Seq(GSE14097)/Homer | 1.00E-03 | 0.0339 | 77.46% |
| 13 | 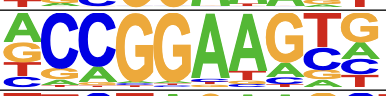 | ETV4(ETS)/HepG2-ETV4-ChIP-Seq(ENCODE)/Homer | 1.00E-03 | 0.0464 | 70.42% |
| 14 | 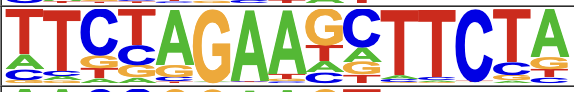 | HSFB4(HSF)/col-HSFB4-DAP-Seq(GSE60143)/Homer | 1.00E-03 | 0.0464 | 8.45% |
